# A multimodal approach to cross-lingual sentiment analysis with ensemble of transformer and LLM

**DOI:** 10.1038/s41598-024-60210-7

**Published:** 2024-04-26

**Authors:** Md Saef Ullah Miah, Md Mohsin Kabir, Talha Bin Sarwar, Mejdl Safran, Sultan Alfarhood, M. F. Mridha

**Affiliations:** 1 Department of Computer Science, American International University-Bangladesh, Dhaka, 1229 Bangladesh; 2 Faculty of Informatics, Eötvös Loránd University, Budapest, 1117 Hungary; 3 Research Chair of Online Dialogue and Cultural Communication, Department of Computer Science, College of Computer and Information Sciences, King Saud University, 11543 Riyadh, Saudi Arabia; 4 Department of Computer Science, College of Computer and Information Sciences, King Saud University, P.O. Box 51178, 11543 Riyadh, Saudi Arabia

**Keywords:** Cross-lingual communication, Sentiment analysis, Neural machine translation, Pretrained sentiment analyzer model, Ensemble with LLM, Computational science, Computer science, Information technology

## Abstract

Sentiment analysis is an essential task in natural language processing that involves identifying a text’s polarity, whether it expresses positive, negative, or neutral sentiments. With the growth of social media and the Internet, sentiment analysis has become increasingly important in various fields, such as marketing, politics, and customer service. However, sentiment analysis becomes challenging when dealing with foreign languages, particularly without labelled data for training models. In this study, we propose an ensemble model of transformers and a large language model (LLM) that leverages sentiment analysis of foreign languages by translating them into a base language, English. We used four languages, Arabic, Chinese, French, and Italian, and translated them using two neural machine translation models: LibreTranslate and Google Translate. Sentences were then analyzed for sentiment using an ensemble of pre-trained sentiment analysis models: Twitter-Roberta-Base-Sentiment-Latest, bert-base-multilingual-uncased-sentiment, and GPT-3, which is an LLM from OpenAI. Our experimental results showed that the accuracy of sentiment analysis on translated sentences was over 86% using the proposed model, indicating that foreign language sentiment analysis is possible through translation to English, and the proposed ensemble model works better than the independent pre-trained models and LLM.

## Introduction

Sentiment analysis, the computational task of determining the emotional tone within a text, has evolved as a critical subfield of natural language processing (NLP) over the past decades^1,2^. It systematically analyzes textual content to determine whether it conveys positive, negative, or neutral sentiments. This capability holds immense importance in understanding public opinion, customer feedback, and social discourse, making it a fundamental principle in various applications across fields such as marketing, politics, and customer service^3–5^. The general area of sentiment analysis has experienced exponential growth, driven primarily by the expansion of digital communication platforms and massive amounts of daily text data. However, the effectiveness of sentiment analysis has primarily been demonstrated in English owing to the availability of extensive labelled datasets and the development of sophisticated language models^6^. This leaves a significant gap in analysing sentiments in non-English languages, where labelled data are often insufficient or absent^7,8^.

Despite the growth in sentiment analysis research, a significant unanswered question persists: How can we effectively adapt sentiment analysis techniques to non-English languages without substantial labelled data? Our study seeks a persuasive answer to this question by presenting a comprehensive methodology and empirical results that demonstrate the feasibility and accuracy of cross-lingual sentiment analysis through translation.

This study explores translating foreign languages into a base language, English, to analyze text sentiments. For sentiment analysis, there are several advantages to translating foreign languages into a base language, such as English. These include:

- *Overcoming language barriers* Language barriers pose a significant challenge in analyzing sentiments in foreign languages. By translating foreign text into a base language, such as English, analysts can overcome these barriers and analyze sentiment more accurately.
- *Standardization of language* Translating foreign languages to a base language can help standardize the language used for sentiment analysis. This can reduce the variability in the language used in different languages and make it easier to compare sentiment across different texts.
- *Availability of sentiment analysis tools* Many sentiment analysis tools are available in English, which makes it easier to analyze sentiment in the translated text. Analysts can use these tools to analyze sentiments in translated texts more efficiently and accurately.
- *Improved accuracy* Translating foreign languages into a base language can improve the accuracy of sentiment analysis compared with traditional sentiment analysis approaches that do not consider language translation. This is because language translation can capture the nuances of foreign languages and convey them more easily to analysts.

The key contributions of this research are outlined as follows:

- Advancement in sentiment analysis research: This study contributes to existing research on sentiment analysis by proposing and implementing a methodology for translating foreign languages into English and analyzing the sentiment of the translated text. This approach expands the scope of sentiment analysis beyond English texts and provides a framework for analyzing sentiments in various languages.
- Insights into the effectiveness of the proposed approach: This study provides insight into the effectiveness of the developed methodology for sentiment analysis and language translation. By presenting the methodology and discussing its implementation, this research offers valuable information on the accuracy and reliability of sentiment analysis in foreign languages.
- Methodological contribution: This study describes a methodology for translating foreign languages into English and conducting sentiment analysis. This contribution includes the techniques, algorithms, and tools employed in the translation process and sentiment analysis, providing a framework for other researchers to replicate or further improve.
- Findings and implications: This study presents findings regarding the sentiment analysis of foreign languages and discusses their implications. These findings shed light on the challenges, limitations, and opportunities associated with sentiment analysis in different languages. These implications are valuable for researchers and practitioners in natural language processing, machine learning, social media analysis, and cross-cultural communication.
- Practical insights for researchers and practitioners: This study aims to provide practical insights that are useful to researchers and practitioners in various domains. The findings and methodology presented in this research can guide future studies in sentiment analysis and language translation. Moreover, practitioners seeking to implement sentiment analysis in multilingual contexts can benefit from the insights and recommendations offered by this study.

By highlighting these contributions, this study demonstrates the novel aspects of this research and its potential impact on sentiment analysis and language translation.

The subsequent sections of this manuscript are structured as follows: In “Related works” section , we delve into the existing body of research relevant to our study. In “Problem formulation” section formulates the problem statement, while “Methodology” section outlines the methodology employed in this research. In “Results and discussion” section presents the experimental results and the accompanying discussions. The challenges of current technologies are presented in the “Challenges” section. Lastly, we draw our conclusions in “Conclusions and future works” section.

## Related works

Sentiment analysis, a crucial natural language processing task, involves the automated detection of emotions expressed in text, distinguishing between positive, negative, or neutral sentiments. The digital age has enabled sentiment analysis across diverse domains. Nonetheless, conducting sentiment analysis in foreign languages, particularly without annotated data, presents complex challenges^9^. While traditional approaches have relied on multilingual pre-trained models for transfer learning, limited research has explored the possibility of leveraging translation to conduct sentiment analysis in foreign languages. Most studies have focused on applying transfer learning using multilingual pre-trained models, which have not yielded significant improvements in accuracy. However, the proposed method of translating foreign language text into English and subsequently analyzing the sentiment in the translated text remains relatively unexplored. This section presents an overview of related works in the field, highlighting the existing studies that have predominantly centred on transfer learning with multilingual pre-trained models and the gaps in testing the effectiveness of the proposed translation-based approach.

The work by Salameh et al.^10^ presents a study on sentiment analysis of Arabic social media posts using state-of-the-art Arabic and English sentiment analysis systems and an Arabic-to-English translation system. This study outlines the advantages and disadvantages of each method and conducts experiments to determine the accuracy of the sentiment labels obtained using each technique. The results show that the sentiment analysis of English translations of Arabic texts produces competitive results. The study also answers several research questions related to sentiment prediction accuracy, loss of predictability when translating Arabic text into English, and the accuracy of automatic sentiment analysis compared to human annotation.

The work in^11^, systematically investigates the translation to English and analyzes the translated text for sentiment within the context of sentiment analysis. Arabic social media posts were employed as representative examples of the focus language text. The study reveals that sentiment analysis of English translations of Arabic texts yields competitive results compared with native Arabic sentiment analysis. Additionally, this research demonstrates the tangible benefits that Arabic sentiment analysis systems can derive from incorporating automatically translated English sentiment lexicons. Moreover, this study encompasses manual annotation studies designed to discern the reasons behind sentiment disparities between translations and source words or texts. This investigation is of particular significance as it contributes to the development of automatic translation systems. This research contributes to developing a state-of-the-art Arabic sentiment analysis system, creating a new dialectal Arabic sentiment lexicon, and establishing the first Arabic-English parallel corpus. Significantly, this corpus is independently annotated for sentiment by both Arabic and English speakers, thereby adding a valuable resource to the field of sentiment analysis.

The work described in^12^ focuses on scrutinizing the preservation of sentiment through machine translation processes. To this end, a sentiment gold standard corpus featuring annotations from native financial experts was curated in English. Subsequently, this gold standard corpus was translated into a target language (German) employing a human translator and three distinct machine translation engines (Microsoft, Google, and Google Neural Network) and seamlessly integrated into Geofluent to facilitate pre- and post-processing procedures. Two critical experiments were conducted in this study. The first objective was to assess the overall translation quality using the BLEU algorithm as a benchmark. The second experiment identified which machine translation engines most effectively preserved sentiments. The findings of this investigation suggest that the successful transfer of sentiment through machine translation can be accomplished by utilizing Google and Google Neural Network in conjunction with Geofluent. This achievement marks a pivotal milestone in establishing a multilingual sentiment platform within the financial domain. Future endeavours will further integrate language-specific processing rules to enhance machine translation performance, thus advancing the project’s overarching objectives.

The work described in^13^, introduces GLUECoS, a benchmark designed to assess the efficacy of code-switched natural language processing (NLP) models across diverse tasks, with a particular focus on sentiment analysis. To evaluate sentiment analysis performance, this study employs English-Spanish and English-Hindi datasets, employing a range of cross-lingual embedding techniques such as MUSE, BiCVM, and BiSkip, along with the utilization of multilingual BERT (mBERT). Additionally, the authors proposed a refined version of the mBERT model, which undergoes further fine-tuning on synthetically generated code-switched data to enhance its suitability for code-switched settings. These findings reveal notable advancements in sentiment analysis. Specifically, on the English-Hindi dataset (SAIL), the state-of-the-art (SOTA) F1 score registers at 56.9, while leveraging the modified mBERT model yields the highest F1 score of 59.35. Similarly, for the English-Spanish dataset (Twitter sentiment), the SOTA F1 score was 64.6, with the modified mBERT model achieving the best score of 69.31. These outcomes underscore the efficacy of fine-tuning mBERT on synthetic code-switched data, demonstrating its capability to further optimize multilingual models for code-switching tasks, thereby showcasing promising avenues for enhancing sentiment analysis in code-switched contexts.

**Table 1:** Comparison of studies in sentiment analysis applications in various domains.

Study	Advantages	Limitations
Wahidur et al.^14^	This paper presents a significant advancement in sentiment analysis within cryptocurrency by implementing fine-tuning techniques on large language models. By achieving a remarkable 40% average gain in zero-shot sentiment analysis performance, the study underscores the potential of fine-tuning strategies to optimize the effectiveness of pre-trained models. Moreover, the exploration of instruction-based fine-tuning reveals its efficacy, particularly in enhancing accuracy for larger models, thus providing valuable insights for improving decision-making processes in cryptocurrency investments.	Despite its notable contributions, the paper faces limitations concerning the generalization of findings, particularly for smaller-scale models. The observed reduction in generalization due to complete model capacity utilization highlights potential challenges in applying fine-tuning techniques universally across different model sizes. Additionally, while the study emphasizes the importance of instruction clarity in fine-tuning processes, it reveals the negative impact of longer and more complex instructions on model accuracy, suggesting further investigation into optimizing instruction design to mitigate such limitations.
Xing^15^	This paper pioneers a paradigm shift in financial sentiment analysis (FSA) by exploring the effectiveness of utilizing large language models (LLMs) without fine-tuning. Rooted in Minsky’s theory of mind and emotions, the study proposes a novel design framework, Heterogeneous LLM Agents for FSA (HAD), involving specialized agents instantiated with prior domain knowledge of FSA error types. The framework demonstrates improved accuracies through comprehensive evaluations of FSA datasets, mainly when substantial discussions are generated. This approach bridges the performance gap between naive prompting and fine-tuning. It offers a computationally efficient alternative, paving new avenues for leveraging LLMs in FSA without extensive model training.	First, scalability poses a challenge, as predicting or discussing with LLM agents incurs higher computational costs than traditional statistical analysis methods. While the framework shows promise, further exploration is needed to address scalability concerns, significantly when scaling up the system with more agents. Secondly, the confidentiality of evaluation datasets raises potential issues, particularly regarding the exposure of datasets to LLMs in previous iterations, which could lead to information leaks and biased evaluations. Additionally, while the study effectively identifies and addresses error types in FSA, further investigation is needed to understand the reasons for new or remaining errors made by LLMs and assess human-level performances on FSA datasets. Despite these limitations, the study highlights the versatile capabilities of LLMs in addressing challenging tasks like FSA and calls for continued research in this vital domain, especially in light of advancing artificial general intelligence (AGI) capabilities.
Xu et al.^16^	This study introduces a groundbreaking approach to semantic analysis of medical texts by harnessing the capabilities of large language models (LLMs), exemplified by GPT-4, to enhance similarity metrics for highly specialized domains such as radiology. Through a novel framework, LLMs are employed for zero-shot text identification and label generation, which are then utilized as measurements for text similarity in radiology reports. By testing this framework on the MIMIC dataset, the study demonstrates significant improvements in semantic similarity assessment compared to traditional NLP metrics like ROUGE and BLEU. The proposed methodology surpasses lexical comparison metrics and showcases the potential of AI-driven approaches to revolutionize healthcare informatics by facilitating a deeper understanding of clinical documentation, ultimately advancing precision medicine and evidence-based clinical practices.	One primary limitation lies in the hypothesized “human-in-the-loop” (HITL) component of the methodology, where direct involvement of medical professionals in refining AI-generated labels was not implemented within the scope of the current research. This absence potentially impacts the AI-generated labels’ accuracy, relevance, and clinical utility, highlighting a gap between envisioned capabilities and realized outcomes. Furthermore, the scope of the research is confined to chest X-ray radiology reports, limiting the generalizability of findings across other medical documentation types and specialities. Future research should focus on operationalizing the HITL framework and extending the analysis to encompass a broader spectrum of medical texts, ensuring the relevance and applicability of the proposed method in diverse healthcare informatics applications. By addressing these limitations, subsequent research can build upon the foundational work presented in this study, further enhancing the integration of AI in medical text analysis and advancing precision medicine practices.
Uddin et al.^17^	This paper presents an innovative approach to phishing email detection using a fine-tuned DistilBERT model, achieving high accuracy rates in distinguishing phishing emails from legitimate ones. By addressing class imbalance issues and employing Explainable-AI techniques like LIME and Transformer Interpret, the model’s decision-making process becomes transparent, enhancing user trust and comprehension. This research underscores the potential of advanced language models to improve cybersecurity measures against phishing threats significantly.	Despite its advancements, this study lacks direct human expert involvement in refining AI-generated labels, potentially impacting the model’s clinical validity. Additionally, its focus solely on phishing email detection limits generalizability. Future research should integrate “human-in-the-loop” methodologies and explore broader applications across domains and datasets to ensure robustness and effectiveness.
Rehan et al.^18^	This paper introduces a pioneering framework for multilingual threatening content detection (MTCD) in English and Urdu, addressing a significant gap in the literature concerning low-resource languages. Leveraging transfer learning and fine-tuning techniques, the study explores joint multi-lingual and joint-translated methodologies using state-of-the-art RoBERTa and MuRIL transformer models. The proposed framework achieves benchmark performance, outperforming baselines with 92% accuracy and 90% macro F1-score, particularly excelling with the joint multi-lingual approach. By providing insights into effective multi-lingual NLP frameworks for social media content classification, the research substantially enhances the automated detection of threatening expressions, promoting peace and harmony online.	First, the evaluation is confined to only two languages (English and Urdu), leaving out other low-resource languages that may also require attention for threatening content detection. Expanding the framework to include languages like Russian, Chinese, or Hindi could enhance its practical utility and generalizability. Secondly, while achieving impressive results on a semi-supervised corpus, testing on a larger dataset could provide further insights and ensure the framework’s robustness across diverse linguistic contexts. Additionally, the binary classification approach adopted here might oversimplify the task; future research could explore more nuanced classifications, such as identifying the targeted individuals or communities within threatening content, to enhance the framework’s relevance and effectiveness in real-world applications.

Recent advancements in machine translation have sparked significant interest in its application to sentiment analysis. The work mentioned in^19^ delves into the potential opportunities and inherent limitations of machine translation in cross-lingual sentiment analysis. The crux of sentiment analysis involves acquiring linguistic features, often achieved through tools such as part-of-speech taggers and parsers or fundamental resources such as annotated corpora and sentiment lexica. The motivation behind this research stems from the arduous task of creating these tools and resources for every language, a process that demands substantial human effort. This limitation significantly hampers the development and implementation of language-specific sentiment analysis techniques similar to those used in English. The critical components of sentiment analysis include labelled corpora and sentiment lexica. This study systematically translated these resources into languages that have limited resources. The primary objective is to enhance classification accuracy, mainly when dealing with available (labelled or raw) training instances. In cases where access to training data is constrained, this research explores methods for translating sentiment lexica into the target language while simultaneously striving to enhance machine translation performance by generating additional contextual information.

The experiments conducted in this study focus on both English and Turkish datasets, encompassing movie and product reviews. The classification task involves two-class polarity detection (positive-negative), with the neutral class excluded. Encouraging outcomes are achieved in polarity detection experiments, notably by utilizing general-purpose classifiers trained on translated corpora. However, it is underscored that the discrepancies between corpora in different languages warrant further investigation to facilitate more seamless resource integration.

Additionally, quantitative evidence highlights the intricacies associated with lexica translation, as the inherent differences in expressing sentiment between languages pose challenges in preserving the sentiment of words and phrases during translation processes. This study provides valuable insights into the evolving landscape of cross-lingual sentiment analysis, shedding light on the potential and complexities of leveraging machine translation.

The work in^20^ proposes a solution for finding large annotated corpora for sentiment analysis in non-English languages by utilizing a pre-trained multilingual transformer model and data-augmentation techniques. The authors showed that using machine-translated data can help distinguish relevant features for sentiment classification better using SVM models with Bag-of-N-Grams. The data-augmentation technique used in this study involves machine translation to augment the dataset. Specifically, the authors used a pre-trained multilingual transformer model to translate non-English tweets into English. They then used these translated tweets as additional training data for the sentiment analysis model. This simple technique allows for taking advantage of multilingual models for non-English tweet datasets of limited size.

Table 1 compares five latest works related to sentiment analysis and machine translation. Each study addressed specific aspects and challenges in sentiment analysis across various languages, shedding light on the advantages and limitations of machine translation techniques. The table concisely compares five recent studies in different domains, showing the advantages and limitations of utilizing advanced language models. These studies cover topics ranging from sentiment analysis in cryptocurrency to phishing email detection, highlighting the diverse applications and challenges associated with Large Language Models (LLMs) in various fields. Our study offers a novel solution to the challenges of sentiment analysis across multiple foreign languages. By introducing an ensemble model that combines a transformer and a large language model, our research demonstrates improved accuracy and reliability in sentiment analysis compared with individual pre-trained models or LLMs alone. Moreover, the proposed methodology for translating foreign languages to English before conducting sentiment analysis provides valuable insights into cross-lingual sentiment analysis techniques, with practical implications for business, social media analysis, and government intelligence. Overall, this study significantly advances the field of sentiment analysis by addressing the complexities of sentiment analysis in foreign languages and providing a robust framework for cross-lingual sentiment analysis that can be applied across diverse linguistic contexts.

## Problem formulation

The problem addressed in this study can be formalized as follows. Let Sentiment Analysis be denoted as *SA*, a task in natural language processing (NLP). *SA* involves classifying text into different sentiment polarities, namely positive (P), negative (N), or neutral (U). With the increasing prevalence of social media and the Internet, *SA* has gained significant importance in various fields such as marketing, politics, and customer service. However, sentiment analysis becomes challenging when dealing with foreign languages, particularly without labelled data for training models.

Considering hypothesis *H*, foreign language sentiment analysis is feasible by translating the text into English and analyzing the sentiments in the translated text. We conducted experiments to validate this hypothesis using four different languages: Arabic (A), Chinese (C), French (F), and Italian (I). The translation process usesd the LibreTranslate API (T_libre) and Google Translate API (T_google). Each sentence *s* is then analyzed for sentiment using two pre-trained sentiment analysis models: Twitter-Roberta-Base-Sentiment-Latest (M_Twitter) and Bertweet-Base-Sentiment-Analysis (M_Bertweet) and an ensemble model consisting of Twitter-Roberta-Base-Sentiment-Latest^21^, bert-base-multilingual-uncased-sentiment^22^, and GPT-3^23^.

To measure the accuracy of sentiment analysis on translated sentences, we define *Acc* as an accuracy metric. *Acc* is the ratio of correctly classified sentences to the total number of sentences analyzed. Mathematically, *Acc* is given by the Eq. (1):

1 $$\begin{aligned} Acc = (C_{correct} / C_{total}) * 100 \end{aligned}$$

$C\_correct$ represents the count of correctly classified sentences, and $C\_total$ denotes the total number of sentences analyzed.

The primary objective of this study is to assess the feasibility of sentiment analysis of translated sentences, thereby providing insights into the potential of utilizing translated text for sentiment analysis and developing a new model for better accuracy. By evaluating the accuracy of sentiment analysis using *Acc*, we aim to validate hypothesis *H* that foreign language sentiment analysis is possible through translation to English.

The results of this study have implications for cross-lingual communication and understanding. If Hypothesis *H* is supported, it would signify the viability of sentiment analysis in foreign languages, thus facilitating improved comprehension of sentiments expressed in different languages. The findings of this research can be valuable into various domains, such as multilingual marketing campaigns, cross-cultural analysis, and international customer service, where understanding sentiment in foreign languages is of utmost importance.

## Methodology

**Figure 1:**
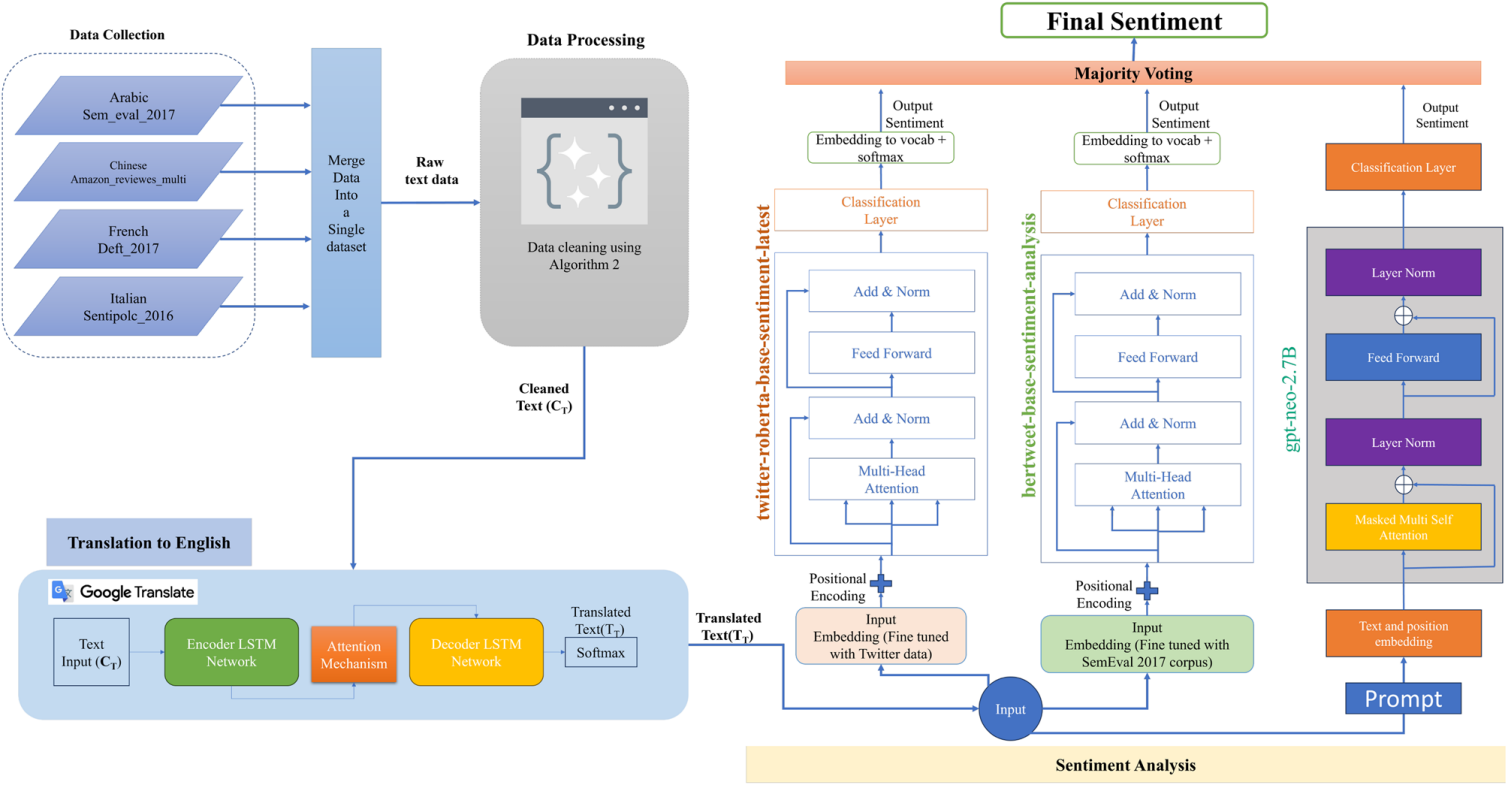
Overview of the proposed method.

In this study, we employed a multi-step methodology to analyze the sentiment of foreign language text by translating them to a base language, English. The methodology comprises five phases: data collection, data cleaning and pre-processing, translation to English, sentiment analysis, and result evaluation. First, we collected data in the target language from various sources such as social media, news articles, and online forums. Next, we performed data cleaning and pre-processing to remove noise, duplicate content, and irrelevant information from the data. Afterwards, we translated the cleaned and pre-processed data into English using a machine translation system. Then, we analyzed the translated data using a sentiment analysis model designed for English text. Finally, we evaluated the results of sentiment analysis to determine the accuracy and effectiveness of the approach. Figure 1 shows the overview of the methodology employed in this study. In the following sections, we provide detailed descriptions of each phase of the methodology and the tools and techniques used in each phase.

### Data collection

**Figure 2:**
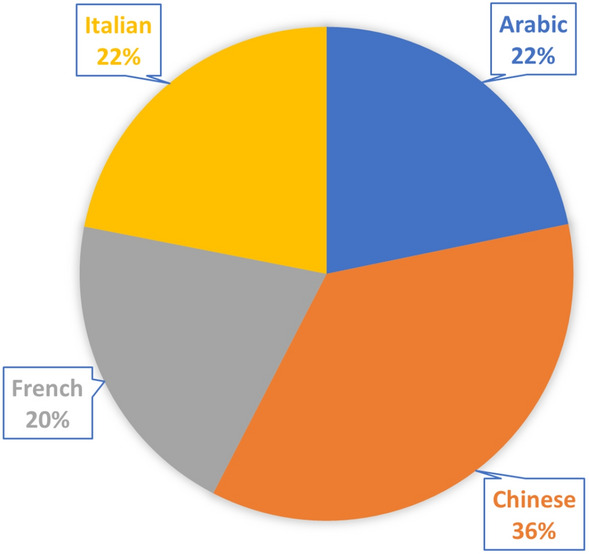
Distribution of different languages in the dataset used in this study.

In the initial stage of the research methodology, data in the target language was gathered from diverse and well-established sources, including SemEval-2017 Task 4: Sentiment Analysis in Twitter^24^, amazon_reviews_multi^25^, DEFT 2017^26^, and SENTIPOLC 2016^27^. This study employs four different languages, namely, Arabic (ar), Chinese (zh), French (fr), and Italian (it), which were selected based on their frequent usage in tweets on the Twitter platform. Additionally, the selection of these languages is supported by studies^28,29^, which have demonstrated that they are among the most commonly used message languages on Twitter. Moreover, these languages are among the most widely spoken languages in the world^30^. Each dataset has been annotated with three sentiment labels, namely, “positive”, “negative”, and “neutral”. These annotation tasks have taken place manually by the crowd workers in crowd-sourcing platforms. Table 2 shows the overview of the data collected and employed in this study. This table presents the data source, language, and number of sentences per data source employed in this study. Figure 2 shows the distribution of different languages in the utilized dataset.

**Table 2:** Overview of the data employed in this study.

Data source	Language	Number of datapoints
SemEval-2017 Task 4	Arabic (ar)	1823
amazon_reviews_multi	Chinese (zh)	3000
DEFT 2017	French (fr)	1715
SENTIPOLC 2016	Italian (it)	1837

### Data cleaning and pre-processing

In the second phase of the methodology, the collected data underwent a process of data cleaning and pre-processing to eliminate noise, duplicate content, and irrelevant information. This process involved multiple steps, including tokenization, stop-word removal, and removal of emojis and URLs. Tokenization was performed by dividing the text into individual words or phrases. In contrast, stop-word removal entailed the removal of commonly used words such as “and”, “the”, and “in”, which do not contribute to sentiment analysis. While stemming and lemmatization are helpful in some natural language processing tasks, they are generally unnecessary in Transformer-based sentiment analysis, as the models are designed to handle variations in word forms and inflexions. Therefore, stemming and lemmatization were not applied in this study’s data cleaning and pre-processing phase, which utilized a Transformer-based pre-trained model for sentiment analysis. Emoji removal was deemed essential in sentiment analysis as it can convey emotional information that may interfere with the sentiment classification process. URL removal was also considered crucial as URLs do not provide relevant information and can take up significant feature space. The complete data cleaning and pre-processing steps are presented in Algorithm 1.

**Algorithm 1:**
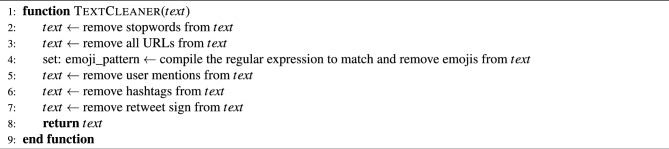
Text Cleaner Algorithm

### Translation to base language: English

In the third phase of the methodology, we translated the cleaned and pre-processed data to English using a self-hosted machine translation system, namely LibreTranslate^31^ and a cloud-hosted service by Google translate neural machine translation (NMT)^32^. LibreTranslate is a free and open-source machine translation API that uses pre-trained NMT models to translate text between different languages. The input text is tokenized and then encoded into a numerical representation using an encoder neural network. The encoded representation is then passed through a decoder network that generates the translated text in the target language. Google Translate NMT uses a deep-learning neural network to translate text from one language to another. The neural network is trained on massive amounts of bilingual data to learn how to translate effectively. During translation, the input text is first tokenized into individual words or phrases, and each token is assigned a unique identifier. The tokens are then fed into the neural network, which processes them in a series of layers to generate a probability distribution over the possible translations. The output from the network is a sequence of tokens in the target language, which are then converted back into words or phrases for the final translated text. The neural network is trained to optimize for translation accuracy, considering both the meaning and context of the input text. One advantage of Google Translate NMT is its ability to handle complex sentence structures and subtle nuances in language.

For the prediction task, the translation process is iterative. Once a sentence’s translation is done, the sentence’s sentiment is analyzed, and output is provided. However, the sentences are initially translated to train the model, and then the sentiment analysis task is performed. The sentiment analysis process is discussed in the following section. Algorithm 2 presents the method employed in this study.

**Algorithm 2:**
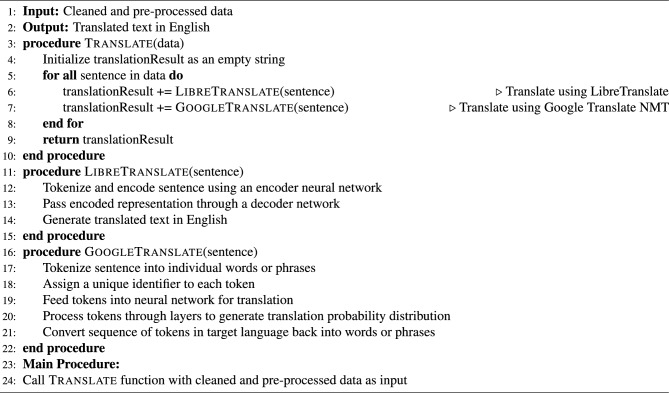
Translation Process

### Sentiment analysis

In the fourth phase of the methodology, we conducted sentiment analysis on the translated data using pre-trained sentiment analysis deep learning models and the proposed ensemble model. In this study, we have utilized an ensemble of two pre-trained sentiment analysis models from Hugging Face^33^, namely, Twitter-Roberta-Base-Sentiment-Latest^34^, bert-base-multilingual-uncased-sentiment^22^ and the GPT-3 LLM from OpenAI^23^. The ensemble sentiment analysis model analyzed the text to determine the sentiment polarity (positive, negative, or neutral). The sentiment analysis process is shown in Algorithm 3. The algorithm shows step by step process followed in the sentiment analysis phase.

Hugging Face is a company that offers an open-source software library and a platform for building and sharing models for natural language processing (NLP). The platform provides access to various pre-trained models, including the Twitter-Roberta-Base-Sentiment-Latest and Bertweet-Base-Sentiment-Analysis models, that can be used for sentiment analysis.

One of the main advantages of using these models is their high accuracy and performance in sentiment analysis tasks, especially for social media data such as Twitter. These models are pre-trained on large amounts of text data, including social media content, which allows them to capture the nuances and complexities of language used in social media^35^. Another advantage of using these models is their ability to handle different languages and dialects. The models are trained on multilingual data, which makes them suitable for analyzing sentiment in text written in various languages^35,36^.

**Algorithm 3:**
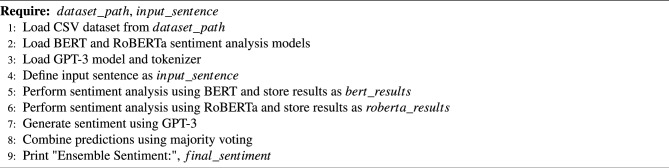
Sentiment Analysis Ensemble Model

The presented algorithm outlines an ensemble model for conducting sentiment analysis by harnessing the capabilities of three distinct natural language processing models: BERT, RoBERTa, and GPT-3. This algorithm begins by importing a CSV dataset and then initializing the sentiment analysis models of BERT and RoBERTa. Additionally, the GPT-3 model and tokenizer are loaded to facilitate the generation of sentiment-related text. The algorithm’s core revolves around an input sentence provided for sentiment analysis. The input sentence is analyzed using the BERT and RoBERTa models, storing their results for further processing. Following this, the GPT-3 model is leveraged to generate the sentiment of the given text based on a fixed prompt “provide the sentiment of the given text in a single class from positive, negative and neutral”.

The next step involves combining the predictions furnished by the BERT, RoBERTa, and GPT-3 models through a process known as majority voting. This entails tallying the occurrences of “positive”, “negative” and “neutral” sentiment labels. Depending on the outcome, and the algorithm ascribes the final sentiment.

### Evaluation metrics

In the final phase of the methodology, we evaluated the results of sentiment analysis to determine the accuracy and effectiveness of the approach. We compared the sentiment analysis results with the ground truth sentiment (the original sentiment of the text labelled in the dataset) to assess the accuracy of the sentiment analysis.

In our evaluation phase, we utilized True Positive (TP), True Negative (TN), False Positive (FP), and False Negative (FN) as metrics for assessing the performance of our binary classifier. These metrics are typically used to evaluate binary classification models. TP represents the number of correctly identified positive instances, TN indicates the number of correctly identified negative instances, FP represents the number of incorrectly classified positive instances, and FN represents the number of incorrectly classified negative instances. Defining TP, TN, FP, and FN is presented in Algorithm 4. This algorithm takes two inputs: the original language sentiment labelled in a dataset and the sentiment after translating it to English. It then compares the two sentiments and determines whether they are positive, negative or neutral. If the original sentiment is positive and the translated sentiment is positive, it is considered a true positive (TP). If the original sentiment is positive and the translated sentiment is negative, it is regarded as a false negative (FN). If the original sentiment is positive and the translated sentiment is neutral, it is considered a false positive (FP). If the original sentiment is negative and the translated sentiment is negative, it is regarded as a true negative (TN). If the original sentiment is negative and the translated sentiment is positive, it is considered a false positive (FP). If the original sentiment is negative and the translated sentiment is neutral, it is regarded as a false negative (FN). If the original and translated sentiments are neutral, it is considered a true positive (TP). If the original sentiment is neutral and the translated sentiment is positive, it is regarded as a false positive (FP). If the original sentiment is neutral and the translated sentiment is negative, it is considered a false negative (FN).

**Algorithm 4:**
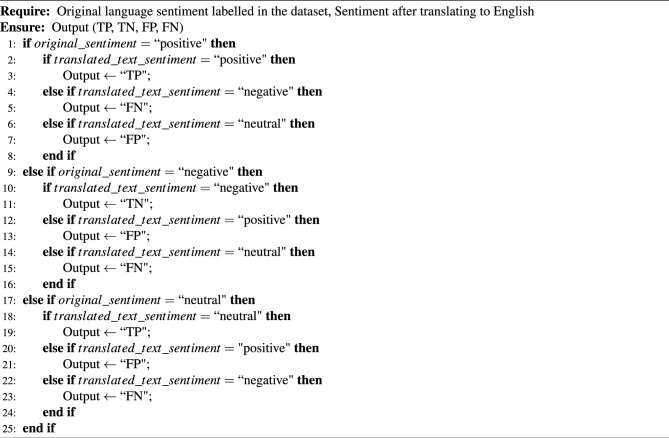
Defining TP, TN, FP and FN

Our study has three distinct classes: positive, negative, and neutral. As a result, we computed the metrics for each class separately. Precision is used to measure the ratio of correctly classified instances among all the instances the classifier identified as positive, while recall measures the proportion of correctly identified instances among all the positive instances in the dataset. The F1-score is the harmonic mean of precision and recall. Accuracy measures the ratio of correctly classified instances among all instances in the dataset. In contrast, specificity measures the proportion of correctly classified negative instances among all negative instances in the dataset. The evaluation metrics can be expressed by the following Eqs. (2), (3), (4), (5), and (6).

2 $$\begin{aligned}{} & {} Precision = \frac{TP}{TP + FP} \end{aligned}$$

3 $$\begin{aligned}{} & {} Recall = \frac{TP}{TP + FN}\end{aligned}$$

4 $$\begin{aligned}{} & {} F1 = 2 \cdot \frac{Precision \cdot Recall}{Precision + Recall}\end{aligned}$$

5 $$\begin{aligned}{} & {} Accuracy = \frac{TP + TN}{TP + TN + FP + FN} \end{aligned}$$

6 $$\begin{aligned}{} & {} Specificity = \frac{TN}{TN + FP} \end{aligned}$$

## Results and discussion

In this section, we present and discuss the results of our experiment on sentiment analysis of foreign languages using machine learning models. We tested two different translation services, namely LibreTranslate and Google Translate, to translate Arabic, Chinese, French, and Italian sentences into English. The translated sentences were then analyzed for sentiment using three different pre-trained sentiment analysis models: Twitter-RoBERTa-Base-Sentiment-Latest, BERTweet-Base-Sentiment-Analysis, GPT-3, which is an LLM, and the proposed ensemble model. We conducted 8 experiments for each language pair, resulting in 32 experiments. We present the results of these experiments and discuss the performance of the translation services and the sentiment analysis models. Finally, we conclude the feasibility and effectiveness of using translated foreign language sentences for sentiment analysis.

**Table 3:** Experimental results for different combinations of translator and sentiment analyzer model.

Translator	Sentiment analyzer model	Accuracy	Precision	Recall	F1	Specificity
LibreTranslate	Twitter-roberta-base	0.5531	0.6440	0.6069	0.6249	0.4678
	Bertweet-base	0.5638	0.6555	0.6155	0.6349	0.4807
	GPT-3	0.8091	0.6909	0.7081	0.6993	0.4522
	Proposed ensemble model	0.8491	0.7892	0.7771	0.7831	0.5201
Google translate	Twitter-roberta-base	0.6182	0.6982	0.6521	0.6744	0.5660
	Bertweet-base	0.6347	0.7066	0.6734	0.6896	0.5761
	GPT-3	0.8571	0.7053	0.7366	0.7199	0.4706
	Proposed ensemble model	0.8671	0.8091	0.8122	0.8106	0.5713

Table 3 showcases the outcomes of diverse combinations involving translator and sentiment analyzer models on sentiment analysis tasks. The presented metrics encompass Accuracy, Precision, Recall, F1 Score, and Specificity, collectively offering a comprehensive assessment of the performance of various combinations in sentiment analysis tasks. The evaluation encompasses two primary translation services, namely LibreTranslate and Google Translate, coupled with four distinct sentiment analyzer models: Twitter-Roberta-Base, Bertweet-Base, GPT-3, and a novel Proposed Ensemble model.

Regarding accuracy, it is notable that the LibreTranslate-Bertweet-Base combination exhibited the lowest accuracy score of 0.5638 across all tested combinations. Conversely, Google Translate, combined with the Proposed Ensemble model, yielded the highest accuracy score of 0.8671, demonstrating its potential for achieving superior sentiment analysis results.

The performance of the GPT-3 model is noteworthy, as it consistently demonstrated strong sentiment analysis capabilities when paired with either the LibreTranslate or Google Translate services. This finding underscores the versatility and robustness of the GPT-3 model for sentiment analysis tasks across different translation platforms.

**Figure 3:**
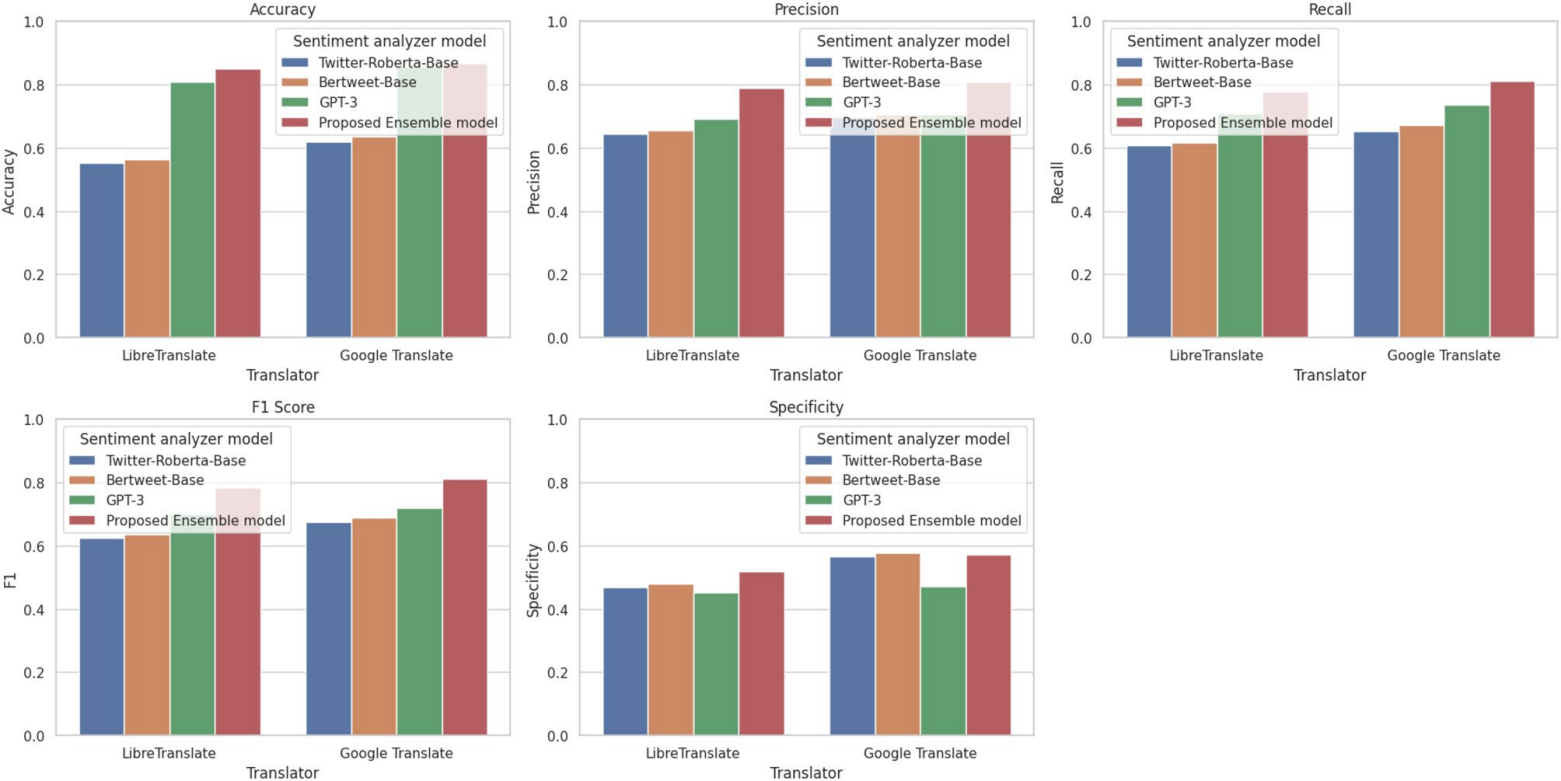
Experimental results showing the outcomes of different evaluation metrics.

Moreover, the Proposed Ensemble model consistently delivered competitive results across multiple metrics, emphasizing its effectiveness as a sentiment analyzer across various translation contexts. This observation suggests that the ensemble approach can be valuable in achieving accurate sentiment predictions. A series of graphs have been generated to visually represent the experimental outcomes of various combinations of translators and sentiment analyzer models, offering a comprehensive insight into the effectiveness of these models in sentiment analysis shown in Fig. 3.

In the Accuracy Graph (Top Left), each graph represents the accuracy achieved by different combinations of translators and sentiment analyzer models. The x-axis identifies the translator used, while the y-axis denotes the accuracy score. The Google Translate combined with the proposed Ensemble Model emerges as the most accurate, yielding an accuracy score of approximately 0.8671.

Moving to the Precision Graph (Top Middle), this visualization focuses on precision, which measures the model’s ability to correctly identify instances of positive sentiment. Within this graph, Google Translate combined with the proposed Ensemble Model stands out with the highest precision score, reaching around 0.8091.

The Recall Graph (Top Right) centres on the recall metric, indicating the model’s proficiency in identifying all relevant positive sentiment instances. Here, Google Translate, paired with the proposed Ensemble Model, demonstrates the highest recall, approximately 0.8122.

The F1 Score Graph (Bottom Left) provides an overview of the F1 score, a metric that balances precision and recall to gauge the overall effectiveness of the sentiment analysis models. In this graph, Google Translate and the proposed Ensemble Model showcase the highest F1 score, at approximately 0.8106.

The Specificity Graph (Bottom Middle) focuses on specificity, a critical metric in sentiment analysis that assesses the model’s capacity to identify negative sentiment accurately. Here, Google Translate paired with the proposed Ensemble Model exhibits the highest specificity, roughly 0.5713.

**Figure 4:**
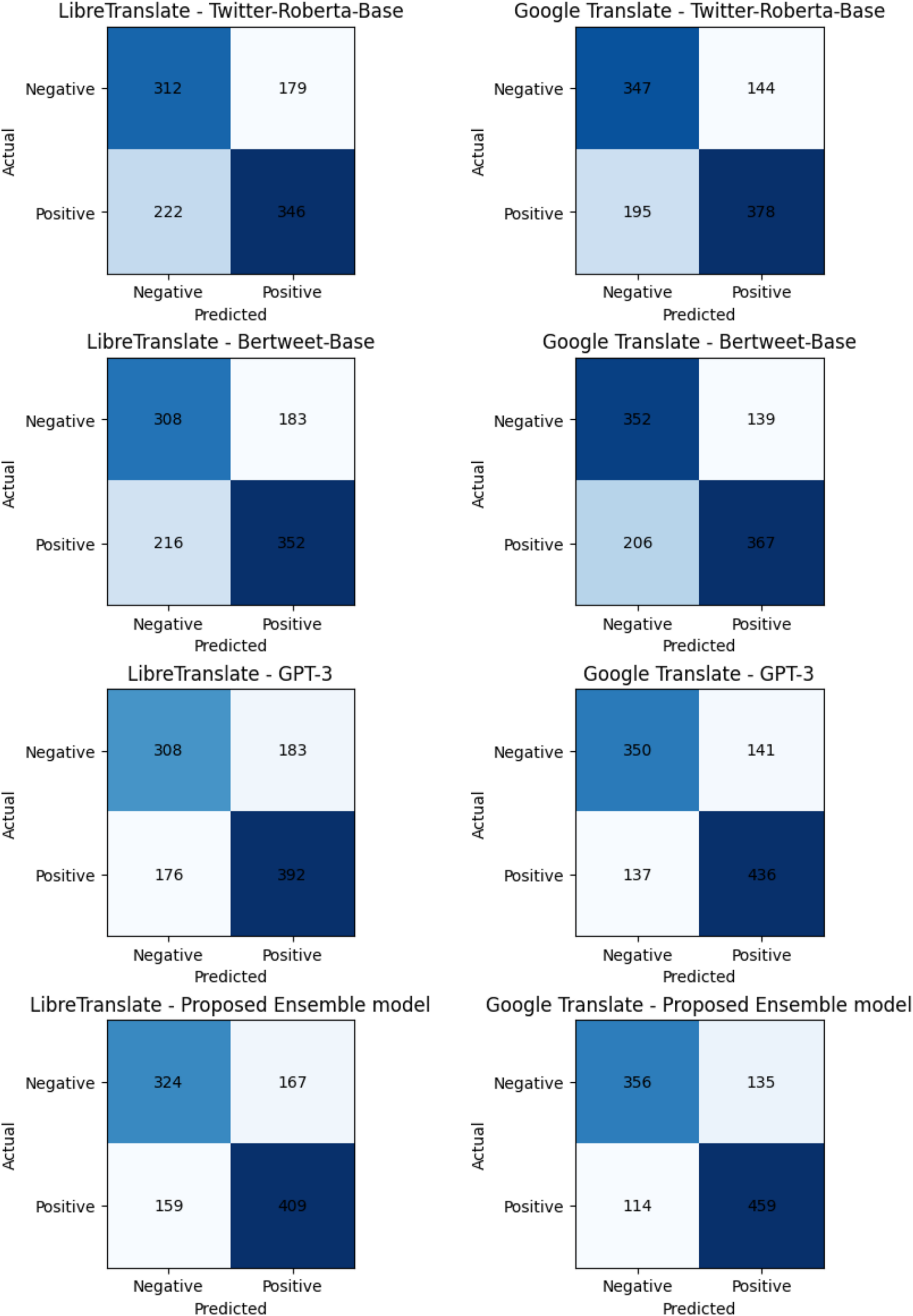
Confusion matrices from different experiments.

The confusion matrices shown in Fig. 4 provide a detailed summary of the performance of different translator and sentiment analyzer model combinations in classifying sentiment. Each confusion matrix consists of four quadrants representing the following:

- *True negative (TN)* The number of instances correctly classified as negative sentiment.
- *False positive (FP)* The number of instances incorrectly classified as positive sentiment.
- *False negative (FN)* The number of instances incorrectly classified as negative sentiment.
- *True positive (TP)* The number of instances correctly classified as positive sentiment.

For instance, considering the confusion matrix for the LibreTranslate - Twitter-Roberta-Base combination, it shows that out of 891 instances:

- 312 were correctly classified as negative sentiment (TN).
- 179 were incorrectly classified as positive sentiment when they were actually negative (FP).
- 222 were incorrectly classified as negative sentiment when they were actually positive (FN).
- 346 were correctly classified as positive sentiment (TP).

Similarly, each confusion matrix provides insights into the strengths and weaknesses of different translator and sentiment analyzer model combinations in accurately classifying sentiment. Evaluating the numbers in these matrices helps understand the models’ overall performance and effectiveness in sentiment analysis tasks.

These graphical representations serve as a valuable resource for understanding how different combinations of translators and sentiment analyzer models influence sentiment analysis performance. Researchers and practitioners can leverage these visualizations to identify the most effective combinations for their specific applications, be it sentiment analysis in social media, customer reviews, or any other context, ensuring the optimal performance of sentiment analysis models. Following the presentation of the overall experimental results, the language-specific experimental findings are delineated and discussed in detail below.

Table 4 presents accuracy scores for different combinations of translator and sentiment analyzer models across four languages: Arabic, Chinese, French, and Italian. Across both LibreTranslate and Google Translate, the proposed ensemble model consistently achieves the highest accuracy scores, with values ranging from 0.83 to 0.88 across the four languages. For LibreTranslate, GPT-3 also demonstrates relatively high accuracy, particularly for Arabic, achieving a score of 0.81. Meanwhile, Google Translate paired with GPT-3 or the proposed ensemble model consistently outperforms other combinations, with accuracy scores ranging from 0.84 to 0.88 across languages. Notably, Chinese consistently scores higher accuracy than other languages across various translator and sentiment analyzer combinations. These findings suggest that the ensemble model, along with GPT-3, holds promise for enhancing accuracy in multilingual sentiment analysis tasks, with Chinese being relatively easier to analyze sentiment accurately.

**Table 4:** Language-wise accuracy scores among different combinations of translator and sentiment analyzer model.

Translator	Sentiment anlyser model	Arabic	Chinese	French	Italian
Language-wise accuracy					
LibreTranslate	Twitter-roberta-base	0.55	0.5	0.56	0.63
LibreTranslate	Bertweet-base	0.57	0.5	0.6	0.63
LibreTranslate	GPT-3	0.81	0.78	0.81	0.82
LibreTranslate	Proposed ensemble model	0.83	0.85	0.84	0.83
Google translate	Twitter-roberta-base	0.61	0.61	0.58	0.67
Google translate	Bertweet-base	0.62	0.62	0.62	0.68
Google translate	GPT-3	0.84	0.87	0.84	0.83
Google translate	Proposed ensemble model	0.86	0.88	0.85	0.84

Table 5 provides precision scores for different combinations of translator and sentiment analyzer models across four languages: Arabic, Chinese, French, and Italian. Within the LibreTranslate framework, the proposed ensemble model consistently achieves the highest precision scores across all languages, ranging from 0.75 to 0.82. Notably, the precision scores are relatively higher for Arabic and Chinese than for French and Italian. Similarly, the proposed ensemble model for Google Translate demonstrates superior precision scores, with values ranging from 0.7 to 0.87 across the four languages. Again, Arabic and Chinese exhibit higher precision scores than French and Italian. Additionally, GPT-3 paired with both LibreTranslate and Google Translate consistently shows competitive precision scores across all languages. These findings suggest that the proposed ensemble model, along with GPT-3, holds promise for improving precision in multilingual sentiment analysis tasks across diverse linguistic contexts.

**Table 5:** Language-wise precision scores among different combinations of translator and sentiment analyzer model.

Translator	Sentiment anlyser model	Arabic	Chinese	French	Italian
Language-wise precision					
LibreTranslate	Twitter-roberta-base	0.61	0.61	0.61	0.74
LibreTranslate	Bertweet-base	0.63	0.62	0.64	0.74
LibreTranslate	GPT-3	0.64	0.7	0.65	0.77
LibreTranslate	Proposed ensemble model	0.79	0.82	0.75	0.78
Google translate	Twitter-roberta-base	0.67	0.74	0.61	0.75
Google translate	Bertweet-base	0.67	0.74	0.64	0.76
Google translate	GPT-3	0.68	0.78	0.66	0.7
Google translate	Proposed ensemble model	0.81	0.85	0.7	0.87

Table 6 depicts recall scores for different combinations of translator and sentiment analyzer models. Across both LibreTranslate and Google Translate frameworks, the proposed ensemble model consistently demonstrates the highest recall scores across all languages, ranging from 0.75 to 0.82. Notably, for Arabic, Chinese, and French, the recall scores are relatively higher compared to Italian. Similarly, GPT-3 paired with both LibreTranslate and Google Translate consistently shows competitive recall scores across all languages. For Arabic, the recall scores are notably high across various combinations, indicating effective sentiment analysis for this language. These findings suggest that the proposed ensemble model, along with GPT-3, holds promise for improving recall in multilingual sentiment analysis tasks across diverse linguistic contexts.

**Table 6:** Language-wise recall scores among different combinations of translator and sentiment analyzer model.

Translator	Sentiment anlyser model	Arabic	Chinese	French	Italian
Language-wise recall					
LibreTranslate	Twitter-roberta-base	0.62	0.49	0.67	0.7
LibreTranslate	Bertweet-base	0.65	0.5	0.69	0.68
LibreTranslate	GPT-3	0.69	0.68	0.75	0.7
LibreTranslate	Proposed ensemble model	0.72	0.75	0.78	0.81
Google translate	Twitter-roberta-base	0.69	0.55	0.68	0.72
Google translate	Bertweet-base	0.71	0.58	0.72	0.73
Google translate	GPT-3	0.73	0.7	0.74	0.78
Google translate	Proposed ensemble model	0.79	0.81	0.8	0.82

Table 7 presents F1 scores for different combinations of translator and sentiment analyzer models across four languages: Arabic, Chinese, French, and Italian. Across both LibreTranslate and Google Translate frameworks, the proposed ensemble model consistently achieves the highest F1 scores across all languages, ranging from 0.746 to 0.844. Notably, for Chinese and Italian, the F1 scores are relatively higher than in Arabic and French. Additionally, GPT-3 paired with both LibreTranslate and Google Translate consistently demonstrates competitive F1 scores across all languages. For Chinese, in particular, the F1 scores are notably high across various combinations, indicating effective sentiment analysis for this language. These findings suggest that the proposed ensemble model, along with GPT-3, holds promise for improving F1 scores in multilingual sentiment analysis tasks across diverse linguistic contexts.

**Table 7:** Language wise F1 scores among different combinations of translator and sentiment analyzer model.

Translator	Sentiment anlyser model	Arabic	Chinese	French	Italian
Language-wise F1					
LibreTranslate	Twitter-roberta-base	0.62	0.54	0.64	0.71
LibreTranslate	Bertweet-base	0.63	0.553	0.66	0.71
LibreTranslate	GPT-3	0.66	0.68	0.69	0.73
LibreTranslate	Proposed ensemble model	0.75	0.78	0.76	0.79
Google translate	Twitter-roberta-base	0.68	0.63	0.64	0.74
Google translate	Bertweet-base	0.68	0.65	0.67	0.74
Google translate	GPT-3	0.70	0.73	0.69	0.73
Google translate	Proposed ensemble model	0.79	0.82	0.74	0.84

Table 8 provides specificity scores for different combinations of translator and sentiment analyzer models across four languages: Arabic, Chinese, French, and Italian. Specificity measures the proportion of correctly identified negative instances out of all actual negative instances. Across both LibreTranslate and Google Translate frameworks, the proposed ensemble model consistently achieves the highest specificity scores across all languages, ranging from 0.49 to 0.73. Notably, the specificity scores for Chinese are relatively higher than those for other languages. Additionally, Google Translate paired with the proposed ensemble model demonstrates high specificity scores for Chinese and Italian. However, it’s important to note that specificity scores are generally lower than other evaluation metrics, indicating that some models may struggle to identify negative sentiment instances accurately. These findings suggest that while the proposed ensemble model shows promise for improving specificity in sentiment analysis, further refinement may be needed to enhance performance across all languages and translators.

**Table 8:** Language wise specificity scores among different combinations of translator and sentiment analyzer model.

Translator	Sentiment anlyser model	Arabic	Chinese	French	Italian
Language-wise specificity					
LibreTranslate	Twitter-roberta-base	0.43	0.51	0.4	0.49
LibreTranslate	Bertweet-base	0.44	0.5	0.45	0.5
LibreTranslate	GPT-3	0.43	0.43	0.44	0.5
LibreTranslate	Proposed ensemble model	0.5	0.54	0.49	0.53
Google translate	Twitter-roberta-base	0.48	0.7	0.42	0.55
Google translate	Bertweet-base	0.48	0.69	0.48	0.57
Google translate	GPT-3	0.46	0.54	0.45	0.42
Google translate	Proposed ensemble model	0.54	0.73	0.51	0.52

After that, this dataset is also trained and tested using an eXtended Language Model (XLM), XLM-T^37^. Which is a multilingual language model built upon the XLM-R architecture but with some modifications. Similar to XLM-R, it can be fine-tuned for sentiment analysis, particularly with datasets containing tweets due to its focus on informal language and social media data. However, for the experiment, this model was used in the baseline configuration and no fine tuning was done. Similarly, the dataset was also trained and tested using a multilingual BERT model called mBERT^38^. The experimental results are shown in Table 9 with the comparison of the proposed ensemble model.

The experimental result reveals promising performance gains achieved by the proposed ensemble models compared to established sentiment analysis models like XLM-T and mBERT. Both proposed models, leveraging LibreTranslate and Google Translate respectively, exhibit better accuracy and precision, surpassing 84% and 80%, respectively. Compared to XLM-T’s accuracy of 80.25% and mBERT’s 78.25%, these ensemble approaches demonstrably improve sentiment identification capabilities. The Google Translate ensemble model garners the highest overall accuracy (86.71%) and precision (80.91%), highlighting its potential for robust sentiment analysis tasks. While mBERT exhibits the highest recall (83.27%). The consistently lower specificity across all models underscores the shared challenge of accurately distinguishing neutral text from positive or negative sentiment, requiring further exploration and refinement. Compared to the other multilingual models, the proposed model’s performance gain may be due to the translation and cleaning of the sentences before the sentiment analysis task.

**Table 9:** Comparative results of the experiment with multilingual model.

Model name	Accuracy	Precision	Recall	F1	Specificity
Proposed Ensemble model—LibreTranslate	0.8491	0.7892	0.7771	0.7831	0.5201
Proposed ensemble model—google translate	0.8671	0.8091	0.8122	0.8106	0.5713
XLM-T	0.8025	0.8089	0.8052	0.807	0.6151
mBERT	0.7825	0.7164	0.8327	0.7701	0.5296

The outcomes of this experimentation hold significant implications for researchers and practitioners engaged in sentiment analysis tasks. The findings underscore the critical influence of translator and sentiment analyzer model choices on sentiment prediction accuracy. Additionally, the promising performance of the GPT-3 model and the Proposed Ensemble model highlights potential avenues for refining sentiment analysis techniques. It opens doors for future research in this dynamic field using LLMs.

In this study, we compared the performance of two popular translators, LibreTranslate and Google Translate, in combination with two pre-trained sentiment analysis models Twitter-Roberta-Base and Bertweet-Base, one Large Language Model GPT-3, and two multilingual models (XLM-t and mBERT) in four different languages, Arabic, Chinese, French, and Italian with our proposed ensemble model. Our evaluation was based on four metrics, precision, recall, F1 score, and specificity. Our results indicate that Google Translate, with the proposed ensemble model, achieved the highest F1 score in all four languages. Our findings suggest that Google Translate is better at translating foreign languages into English. The proposed ensemble model is the most suitable option for sentiment analysis on these four languages, considering that different language-translator pairs may require different models for optimal performance.

The results presented in this study provide strong evidence that foreign language sentiments can be analyzed by translating them into English, which serves as the base language. This concept is further supported by the fact that using machine translation and sentiment analysis models trained in English, we achieved high accuracy in predicting the sentiment of non-English languages such as Arabic, Chinese, French, and Italian. The obtained results demonstrate that both the translator and the sentiment analyzer models significantly impact the overall performance of the sentiment analysis task. It opens up new possibilities for sentiment analysis applications in various fields, including marketing, politics, and social media analysis.

## Challenges

Despite the advantages of translating foreign languages to a base language for sentiment analysis, there are several challenges associated with this approach that have surfaced in this experiment and also faced by different studies. This section discusses these challenges in more detail and explores the possible solutions.

### Challenge I: translation accuracy

One of the primary challenges encountered in foreign language sentiment analysis is accuracy in the translation process. Machine translation systems often fail to capture the intricate nuances of the target language, resulting in erroneous translations that subsequently affect the precision of sentiment analysis outcomes^39,40^.

One potential solution to address the challenge of inaccurate translations entails leveraging human translation or a hybrid approach that combines machine and human translation. Human translation offers a more nuanced and precise rendition of the source text by considering contextual factors, idiomatic expressions, and cultural disparities that machine translation may overlook. However, it is essential to note that this approach can be resource-intensive in terms of time and cost. Nevertheless, its adoption can yield heightened accuracy, especially in specific applications that require meticulous linguistic analysis.

Alternatively, machine learning techniques can be used to train translation systems tailored to specific languages or domains. Training the system on extensive datasets and employing specialized machine learning algorithms and natural language processing methodologies can enhance the accuracy of translations, thereby reducing errors in subsequent sentiment analysis. Although it demands access to substantial datasets and domain-specific expertise, this approach offers a scalable and precise solution for foreign language sentiment analysis.

### Challenge II: cultural sensitivity

Another critical consideration in translating foreign language text for sentiment analysis pertains to the influence of cultural variations on sentiment expression. Diverse cultures exhibit distinct conventions in conveying positive or negative emotions, posing challenges for accurate sentiment capture by translation tools or human translators^41,42^.

For instance, certain cultures may predominantly employ indirect means to express negative emotions, whereas others may manifest a more direct approach. Consequently, if sentiment analysis algorithms or models fail to account for these cultural disparities, precisely identifying negative sentiments within the translated text becomes arduous.

To mitigate this concern, incorporating cultural knowledge into the sentiment analysis process is imperative to enhance the accuracy of sentiment identification in translated text. Potential strategies include the utilization of domain-specific lexicons, training data curated for the specific cultural context, or applying machine learning models tailored to accommodate cultural differences. Integrating cultural awareness into sentiment analysis methodologies enables a more refined understanding of the sentiments expressed in the translated text, enabling comprehensive and accurate analysis across diverse linguistic and cultural domains.

### Challenge III: idiomatic expressions

Another challenge when translating foreign language text for sentiment analysis is the idiomatic expressions and other language-specific attributes that may elude accurate capture by translation tools or human translators^43^.

Idioms represent phrases in which the figurative meaning deviates from the literal interpretation of the constituent words. Translating idiomatic expressions can be challenging because figurative connotations may not appear immediately in the translated text.

To proficiently identify sentiment within the translated text, a comprehensive consideration of these language-specific features is imperative, necessitating the application of specialized techniques. For instance, employing sentiment analysis algorithms trained on extensive data from the target language may enhance the capability to discern sentiments within idiomatic expressions and other language-specific attributes. More precise and comprehensive sentiment analysis can be achieved by incorporating techniques explicitly devised to address idiomatic expressions and other language-specific characteristics, thereby facilitating adequate cross-linguistic understanding and analysis.

### Challenge IV: translation biases

An inherent limitation in translating foreign language text for sentiment analysis revolves around the potential introduction of biases or errors stemming from the translation process^44^. Although machine translation tools are often highly accurate, they can generate translations that deviate from the fidelity of the original text and fail to capture the intricacies and subtleties of the source language. Similarly, human translators generally exhibit greater accuracy but are not immune to introducing biases or misunderstandings during translation.

To minimize the risks of translation-induced biases or errors, meticulous translation quality evaluation becomes imperative in sentiment analysis. This evaluation entails employing multiple translation tools or engaging multiple human translators to cross-reference translations, thereby facilitating the identification of potential inconsistencies or discrepancies. Additionally, techniques such as back-translation can be employed, whereby the translated text is retranslated back into the original language and compared to the initial text to discern any disparities. By undertaking rigorous quality assessment measures, the potential biases or errors introduced during the translation process can be effectively mitigated, enhancing the reliability and accuracy of sentiment analysis outcomes.

### Challenge V: language diversity

Another plausible constraint pertains to the practicality and feasibility of translating foreign language text, particularly in scenarios involving extensive text volumes or languages that present significant challenges. Situations characterized by a substantial corpus for sentiment analysis or the presence of exceptionally intricate languages may render traditional translation methods impractical or unattainable^45^. In such cases, alternative approaches are essential to conduct sentiment analysis effectively.

One viable avenue involves the development of language-specific sentiment analysis algorithms tailored to the intricacies of the target language. These algorithms were optimized to address the unique linguistic characteristics, cultural nuances, and sentiment expression patterns specific to the language under consideration. By customizing the sentiment analysis approach, the limitations associated with translation can be circumvented, thereby facilitating accurate sentiment analysis outcomes.

Another approach involves leveraging machine learning techniques to train sentiment analysis models on substantial quantities of data from the target language. This method capitalizes on large-scale data availability to create robust and effective sentiment analysis models. By training models directly on target language data, the need for translation is obviated, enabling more efficient sentiment analysis, especially in scenarios where translation feasibility or practicality is a concern.

By employing these alternative approaches, such as language-specific sentiment analysis algorithms or training on large-scale target language data, the challenges posed by the impractical or unfeasible translation of foreign language text can be effectively addressed, fostering improved sentiment analysis outcomes.

### Challenge VI: handling slang, colloquial language, irony, and sarcasm

One significant challenge in translating foreign language text for sentiment analysis involves incorporating slang or colloquial language, which can perplex both translation tools and human translators^46^. Slang and colloquial languages exhibit considerable variations across regions and languages, rendering their accurate translation into a base language, such as English, challenging. For example, a Spanish review may contain numerous slang terms or colloquial expressions that non-fluent Spanish speakers may find challenging to comprehend. Similarly, a social media post in Arabic may employ slang or colloquial language unfamiliar to individuals who lack knowledge of language and culture. To accurately discern sentiments within text containing slang or colloquial language, specific techniques designed to handle such linguistic features are indispensable.

Another potential challenge in translating foreign language text for sentiment analysis is irony or sarcasm, which can prove intricate in identifying and interpreting, even for native speakers. Irony and sarcasm involve using language to express the opposite of the intended meaning, often for humorous purposes^47,48^. For instance, a French review may use irony or sarcasm to convey a negative sentiment; however, individuals lacking fluency in French may struggle to comprehend this intended tone. Similarly, a social media post in German may employ irony or sarcasm to express a positive sentiment, but this could be arduous to discern for those unfamiliar with language and culture. To accurately identify sentiment within a text containing irony or sarcasm, specialized techniques tailored to handle such linguistic phenomena become indispensable.

Notably, sentiment analysis algorithms trained on extensive amounts of data from the target language demonstrate enhanced proficiency in detecting and analyzing specific features in the text. Another potential approach involves using explicitly trained machine learning models to identify and classify these features and assign them as positive, negative, or neutral sentiments. These models can subsequently be employed to classify the sentiment conveyed within the text by incorporating slang, colloquial language, irony, or sarcasm. This facilitates a more accurate determination of the overall sentiment expressed.

## Conclusions and future works

This study investigated the effectiveness of using different machine translation and sentiment analysis models to analyze sentiments in four foreign languages. Our results indicate that machine translation and sentiment analysis models can accurately analyze sentiment in foreign languages. Specifically, Google Translate and the proposed ensemble model performed the best in terms of precision, recall, and F1 score. Furthermore, our results suggest that using a base language (English in this case) for sentiment analysis after translation can effectively analyze sentiment in foreign languages. This study provides an ensemble model to perform sentiment analysis of foreign languages through machine translation and analysis in a base language, which can have potential applications in various fields, including business, social media analysis, and government intelligence. This model can be extended to languages other than those investigated in this study. We acknowledge that our study has limitations, such as the dataset size and sentiment analysis models used. These limitations should be addressed in future research.

## Data Availability

The datasets generated during and/or analysed during the current study are available from the corresponding author upon reasonable request.
